# Crystal engineering of ionic cocrystals comprising Na/K salts of hesperetin with hesperetin molecules and solubility modulation

**DOI:** 10.1107/S205225252300266X

**Published:** 2023-04-21

**Authors:** Shasha Jin, Molly M. Haskins, Cheng-Hua Deng, Catiúcia R. M. O. Matos, Michael J. Zaworotko

**Affiliations:** a Department of Chemical Sciences and Bernal Institute, University of Limerick, Limerick V94 T9PX, Ireland; University of Iowa, USA

**Keywords:** crystal engineering, ionic cocrystals, phenol–phenolate synthons, nutraceuticals, molecular crystals

## Abstract

Hesperetin (HES) is a weakly acidic flavonoid of topical interest owing to its antiviral properties. Despite the presence of HES in many dietary supplements, its bioavailability is hindered by poor aqueous solubility (1.35 µg ml^−1^) and rapid first-pass metabolism. Cocrystallization has evolved as a promising approach to generate novel crystal forms of biologically active compounds and enhance the physicochemical properties without covalent modification. In this work, crystal engineering principles were employed to prepare and characterize various crystal forms of HES. Specifically, two salts and six new ionic cocrystals (ICCs) of HES involving sodium or potassium salts of HES were studied using single-crystal X-ray diffraction (SCXRD) or powder X-ray diffraction and thermal measurements. Structures of seven of the new crystalline forms were elucidated by SCXRD, which revealed two families of isostructural ICCs in terms of their crystal packing and confirmed the presence of phenol⋯phenolate (PhOH⋯PhO^−^) supramolecular heterosynthons. Diverse HES conformations were observed amongst these structures, including unfolded and folded (previously unreported) conformations. One ICC, HES with the sodium salt of HES (NESNAH), was scalable to the gram scale and found to be stable after accelerated stability testing (exposure to elevated heat and humidity). HESNAH reached *C*_max_ after 10 min in PBS buffer 6.8 compared with 240 min in pure HES. In addition, relative solubility was observed to be 5.5 times greater, offering the possibility of improved HES bioavailability.

## Abstract 2

Synthesis and property evaluation of ionic cocrystals and salts of the weakly acidic nutraceutical compound hesperetin support the reliability of crystal engineering approaches based on the PhOH⋯PhO^−^ supramolecular heterosynthon and the effectiveness of cocrystallization in tackling low aqueous solubility of a weakly ionisable compound.

## 1. Introduction

**Figure 1:**
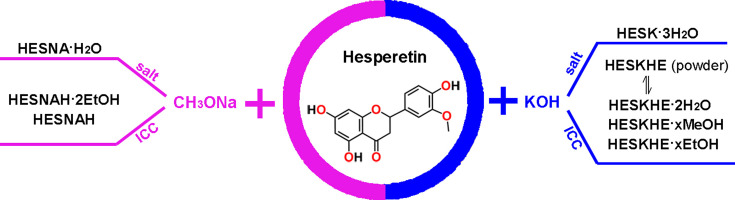
Graphical representation of the salts and ICCs of HES obtained in the study.

Flavonoids feature a polyphenolic backbone and are widely distributed in plants, fruits and vegetables (Panche *et al.*, 2016 ▸). They are widely studied for their beneficial health effects as highlighted in epidemiological studies (Hertog, 1996 ▸; Arts, 2008 ▸). Hesperetin (HES, Fig. 1 ▸), a bioactive flavonoid, is naturally available in citrus fruits and has been formulated into dietary supplements as it possesses potent antioxidant and anti-inflammatory properties (Parhiz *et al.*, 2015 ▸). HES can also play an important protective role in the prevention of various diseases associated with inflammation and oxidative stress, including cancer [*e.g.* carcinoid tumours (Zarebczan *et al.*, 2011 ▸; Sohel *et al.*, 2022 ▸)], neurodegenerative diseases [*e.g.* Alzheimer’s disease (Ikram *et al.*, 2019 ▸; kheradmand *et al.*, 2018 ▸)] and cardiovascular diseases (Roohbakhsh *et al.*, 2015 ▸; Wang *et al.*, 2017 ▸). Most recently, arising from the COVID-19 pandemic, HES has attracted attention because of its ability to bind to multiple regions of severe acute respiratory syndrome coronavirus 2 (SARS-CoV-2), suppressing entry into host cells and subsequent replication of viral particles (Khezri *et al.*, 2022 ▸; Agrawal *et al.*, 2021 ▸). Unfortunately, HES suffers from poor aqueous solubility (1.35 µg ml^−1^, 25°C) (Liu & Chen, 2008 ▸) and undergoes rapid first-pass metabolism (Kanaze *et al.*, 2007 ▸), two factors that can severely limit bioavailability and efficacy. Therefore, improving the solubility and bioavailability of HES is a practical concern for potential pharmacological utility.

In pharmaceutical science, solid-state screening of active pharmaceutical ingredients (APIs), including salts (Berge *et al.*, 1977 ▸; Gould, 1986 ▸; Morris *et al.*, 1994 ▸; Stahl & Wermuth, 2002 ▸), polymorphs (Haleblian & Mccrone, 1969 ▸; Miller *et al.*, 2005 ▸), hydrates/solvates (Healy *et al.*, 2017 ▸; Khankari & Grant, 1995 ▸) and cocrystals (Aitipamula *et al.*, 2012 ▸) is employed to counter undesirable physicochemical properties, enabling the development of bioactive molecules as drug products. Generally, salt formation is the most common and effective way to enhance the solubility of ionizable molecules, with approximately 50% of marketed drug products being in salt form (Byrn *et al.*, 2017 ▸). For weakly ionizable or non-ionizable molecules, where polymorphs and hydrates/solvates tend to not have a significant impact on solubility (Pudipeddi & Serajuddin, 2005 ▸), crystal engineering of pharmaceutical cocrystals has become widely used over the last two decades. Pharmaceutical cocrystals offer an opportunity to control the physicochemical properties of drug molecules (Bolla *et al.*, 2022 ▸), including solubility (Thakuria *et al.*, 2013 ▸; Babu & Nangia, 2011 ▸), hydrolytic stability (Duggirala *et al.*, 2014 ▸) and bioavailability (Nangia & Desiraju, 2022 ▸; Shan *et al.*, 2014 ▸), without changing the molecular structure of an API and thereby compromising its intrinsic biological activities. Indeed, the US Food and Drug Administration (2018 ▸) and European Medicines Agency (2015 ▸) have released regulatory guidelines for industrial use of pharmaceutical cocrystals, *i.e.* cocrystals in which at least one of the coformers is a drug molecule (Almarsson & Zaworotko, 2004 ▸). To date, at least ten drug products whereby pharmaceutical cocrystals serve as drug substances have been approved and brought to market (Kavanagh *et al.*, 2019 ▸; Almansa *et al.*, 2017 ▸).

Cocrystals can be classified as molecular cocrystals (MCCs) comprising two or more molecular compounds (*A* and *B*), or ionic cocrystals (ICCs) (Braga *et al.*, 2010 ▸) comprising at least one salt (*A* ^+^ *B* ^−^C, where *A* ^+^ = cation, *B* ^−^ = anion and *C* = neutral molecule or another salt). Since ICCs include no fewer than three components, at least two components can be changed if one of the coformers is an API or an ionic form of an API, thereby providing more opportunities to modulate the physicochemical properties compared with MCCs, which typically offer only one variable component in addition to the API. Additionally, ICCs comprising the API with its conjugate salt would offer the possibility to increase the drug loading mass, potentially reducing the drug dosage. Cocrystals are typically sustained by hydrogen bonds (Aakeröy & Seddon, 1993 ▸; Bhattacharya & Zaworotko, 2018 ▸) and halogen bonds (Mukherjee *et al.*, 2014 ▸). In the case of ICCs containing metal ions, coordination bonds are likely to be established between organic moieties and metals (Braga *et al.*, 2018 ▸; Grepioni *et al.*, 2022 ▸). Given that coordination bonds are typically stronger than hydrogen bonds, these organic–inorganic assemblies can potentially enhance the relevant properties. Indeed, a particularly relevant example is the marketed drug product Depakote. It is an ICC-containing valproic acid and sodium valproate, which exhibits non-hygroscopicity compared with its parent salt (Petruševski *et al.*, 2008 ▸; Kavanagh *et al.*, 2019 ▸). Likewise, our group demonstrated how ICCs of lithium salicylate with l-proline and lithium halides (Cl^−^, Br^−^) with glucose improve hygroscopicity and can also modulate the pharmacokinetics of parent lithium salts (Duggirala *et al.*, 2014 ▸; Smith *et al.*, 2013 ▸). These ‘organic–inorganic’ ICCs can also be used for solid-state chiral resolution, whereby the lithium cation or zinc cation that favour tetrahedral coordination exhibit homochiral preference when Li*X* (*X* = Cl, Br, I) or ZnCl_2_ are cocrystallized with racemic coformers (Shemchuk *et al.*, 2021 ▸, 2020 ▸, 2018 ▸).

Cocrystals can be rationally designed by crystal engineering based on the exploitation of supramolecular synthons (Desiraju, 1995 ▸). Supramolecular heterosynthons (Walsh *et al.*, 2003 ▸) between two different but complementary functional groups are particularly valuable to facilitate cocrystal design once the relevant hierarchies are established, *e.g.* if a supramolecular heterosynthon is favoured over a competing supramolecular homosynthon between two identical self-complementary functional groups or versus other heterosynthons that might form between coformers (Jin, Sanii *et al.*, 2022 ▸; Haskins *et al.*, 2022 ▸; Duggirala *et al.*, 2015 ▸; Kavuru *et al.*, 2010 ▸; Shattock *et al.*, 2008 ▸; Bis *et al.*, 2007 ▸). In our previous work, we demonstrated that the phenol–phenolate supramolecular heterosynthon (PhOH⋯PhO^−^) is reliable and robust enough to be exploited for the design of ICCs of phenolic compounds (Jin, Sanii *et al.*, 2022 ▸). In this contribution, we report a crystal engineering approach to address the solubility issue of HES through cocrystallization. HES would be expected to form both salts and cocrystals owing to its weakly acidic nature (p*K*_a_ = 6.67) and hydrogen-bonding capabilities, respectively. To date, molecular cocrystals and an ICC of HES with palmatine chloride have been reported (Wang *et al.*, 2021 ▸; Zhang, Yang *et al.*, 2021 ▸; Liu *et al.*, 2022 ▸; Zhang, Zhu *et al.*, 2021 ▸; Zhang *et al.*, 2022 ▸; Chadha *et al.*, 2017 ▸; Kavuru *et al.*, 2010 ▸). A survey of the Cambridge Structural Database using *ConQuest* (version 2020.3.0 with June 2022 update) revealed no salt forms of HES. By introducing pharmaceutically acceptable metal cations, *i.e.* potassium (K^+^) and sodium (Na^+^), here we report the synthesis and characterization of K^+^ and Na^+^ salts of HES as well as ICCs comprising HES and these conjugate salts. We also report the dissolution profiles of scalable ICCs in PBS buffer solution.

## 2. Experimental

### 2.1. Reagents and materials

HES (>97%) and sodium methoxide (*ca* 5 mol l^−1^ in MeOH) were purchased from Tokyo Chemical Industry Co. Ltd. Potassium hydroxide (>85%) was purchased from Sigma–Aldrich. All solvents were purchased from Sigma–Aldrich or Alfa Aesar and used without further purification.

### 2.2. Synthesis

All cocrystallization experiments to isolate single crystals were conducted via slow evaporation, heating–cooling or liquid/vapour diffusion, either at room temperature or in a refrigerator at 5.4°C. Scale-up experiments were conducted by slurrying. Experimental details are as follows.

#### 2.2.1. Hydrated sodium salt of HES, HESNA·H_2_O

Single crystals: HES (30 mg, 0.1 mmol) and 1 *M* sodium methoxide in MeOH (50 µl, 0.05 mmol) were dissolved in 2.3 ml of MeOH in a test tube; 2.5 ml of *n*-hexane was layered on top. The tube was sealed using parafilm and allowed to stand at room temperature. Colourless block crystals of HESNA·H_2_O were isolated after 7 days.

#### 2.2.2. 1:1 ICC of HES and HESNA, HESNAH

Single crystals: HES (30 mg, 0.1 mmol) and 1 *M* sodium methoxide in MeOH (50 µl, 0.05 mmol) were dissolved in 2.3 ml of EtOH in a test tube; 2.5 ml of *n*-hexane was layered on top. The tube was sealed using parafilm and allowed to stand at room temperature. Colourless block crystals of HESNAH were harvested after 2 days.

Scale-up: bulk powder of HESNAH was obtained by slurrying HES (2.0 g, 6.6 mmol) and 5 *M* sodium methoxide in MeOH (700 µl, 3.5 mmol) in 6 ml MeOH (or H_2_O, or EtOH) under ambient conditions for 48 h. The resulting powder was filtered and dried in an oven at 50°C overnight.

#### 2.2.3. Ethanol solvate of HESNAH, HESNAH·2EtOH

Single crystals: HES (30 mg, 0.1 mmol) and 1 *M* sodium methoxide in MeOH (50 µl, 0.05 mmol) were dissolved in 2.5 ml of EtOH in a vial. The vial was uncapped and put in a beaker containing 2.5 ml of *n*-hexane. The beaker was sealed and left at room temperature. After about 6 h, yellowish block crystals of HESNAH·2EtOH were harvested.

#### 2.2.4. Hydrated potassium salt of HES, HESK·3H_2_O

Single crystals: HES (15 mg, 0.05 mmol) and 1 *M* KOH in H_2_O (100 µl, 0.1 mmol) were dissolved in 0.5 ml H_2_O and heated to 120°C for *ca* 15 min in a capped vial on a hot plate. The hot plate was turned off and the vial was allowed to cool on the hot plate. Colourless block crystals of HESK·3H_2_O were harvested after 12 h.

#### 2.2.5. Hydrated 1:1 ICC of HES and HESK, HESKHE·2H_2_O

Single crystals: HES (30 mg, 0.1 mmol) and 1 *M* KOH in H_2_O (100 µl, 0.1 mmol) were dissolved in a vial containing 2.3 ml of an EtOH/H_2_O mixture with a volume ratio of 1:1. The vial was sealed with pierced parafilm to allow the solvent to slowly evaporate at room temperature. Colourless block crystals of HESKHE·2H_2_O were harvested after 3 days.

#### 2.2.6. HESKHE anhydrate

Powder: the powder of HESKHE·2H_2_O was placed in an oven at 160°C for 30 min.

#### 2.2.7. Ethanol solvate of HESKHE, HESKHE·*x*EtOH

Single crystals: HES (30 mg, 0.1 mmol) and 1 *M* KOH in H_2_O (50 µl, 0.05 mmol) were dissolved in 2 ml EtOH in a vial. The vial was sealed with pierced parafilm and left in the fridge at 5.4°C. Colourless block crystals of HESKHE·*x*EtOH were harvested after 3 days.

Scale-up: a bulk sample of HESKHE·*x*EtOH was obtained by slurrying HES (1.5 g, 5 mmol) and solid KOH (151.1 mg, 2.7 mmol) in 6.5 ml EtOH under ambient conditions for 48 h. The resulting powder was filtered and dried in an oven at 50°C overnight.

#### 2.2.8. Methanol solvate of HESKHE, HESKHE·*x*MeOH

HES (30 mg, 0.1 mmol) and 1 *M* KOH in H_2_O (50 µl, 0.05 mmol) were dissolved in 2 ml of MeOH in a test tube; 2.5 ml of *n*-hexane was layered on top. The tube was sealed using parafilm and allowed to stand at room temperature. After 2 days, colourless block crystals of HESKHE·*x*MeOH were harvested.

### 2.3. Thermogravimetric analysis

Thermogravimetric analysis (TGA) was performed on a TA Instruments Q50 TG from room temperature to 350°C at a 10°C min^−1^ heating rate under an N_2_ purge of 60 ml min^−1^.

### 2.4. Differential scanning calorimetry

Thermal analysis was carried out by employing a TA Instruments DSC Q20. Samples were heated in a differential scanning calorimetry (DSC) pan with a pierced lid at a 10°C min^−1^ heating rate under an N_2_ atmosphere.

### 2.5. Powder X-ray diffraction

All powder X-ray diffraction (PXRD) patterns were collected on an Empyrean diffractometer (PANalytical) with the following experimental parameters: Cu *K*α radiation (λ = 1.54056 Å), 40 kV and 40 mA, scan speed 8° min^−1^, step size 0.05°, angle range 5–40° 2θ.

### 2.6. Variable-temperature powder X-ray diffraction

Variable-temperature powder X-ray diffraction (vt-PXRD) was carried out and diffraction patterns were collected using a PANalytical X’Pert Pro-MPD diffractometer equipped with a PIXcel3D detector operating in scanning line detector mode. The diffractometer is outfitted with an Empyrean Cu LFF (long fine-focus) HR (9430 033 7300x) tube operated at 40 kV and 40 mA, and Cu *K*α radiation (λ_α_ = 1.54056 Å). An Anton Paar TTK 450 stage coupled with the Anton Paar TCU 110 Temperature Control Unit was used. Measurements were in continuous scanning mode with the goniometer in the theta–theta orientation. HESNAH and HESKHE·*x*EtOH powders were loaded on a zero-background sample holder made for the Anton Paar TTK 450 chamber. The data were collected in the range 5–40° (2θ) at designated temperature points with a step size of 0.0334225 and a scan time of 50.165 s per step under an N_2_ atmosphere.

### 2.7. Single-crystal X-ray diffraction and structure determination 

Single-crystal structures of HESNA·H_2_O, HESNAH, HESK·3H_2_O, HESKHE·2H_2_O and HESKHE·*x*MeOH were determined using either Mo *K*α (λ = 0.71073 Å) or Cu *K*α (λ = 1.5418 Å) radiation on a Bruker D8 Quest fixed-chi diffractometer equipped with a Bruker APEX-II CCD detector and a nitro­gen-flow Oxford Cryosystem attachment. Data were indexed, integrated and scaled in *APEX3* (Bruker, 2016 ▸). Absorption corrections were performed by the multi-scan method using *SADABS* (Sheldrick, 1996 ▸). Space groups were determined using *XPREP* as implemented in *APEX3*. Single-crystal structures of HESNAH·2EtOH and HESKHE·xEtOH were collected using Mo *K*α radiation (λ = 0.71073 Å) on a Rigaku mm007 Oxford diffractrometer equipped with an R-axis IV++ image plate detector and an Oxford cryosystem 800. All crystal structures were solved using the intrinsic phasing method in *SHELXT* (Sheldrick, 2015*a* ▸) and refined with *SHELXL* (Sheldrick, 2015 ▸ *b*) using the least-squares method implemented in *Olex2* (Dolomanov *et al.*, 2009 ▸). All non-hydrogen atoms were refined anisotropically. Hydrogen atoms of alkyl groups were placed in geometrically calculated positions and included in the refinement process using a riding model (AFIX 137, AFIX 23 or AFIX 13) with isotropic thermal parameters: *U*_iso_ (H) = −1.5*U*_eq_ (–CH_3_), *U*_iso_ (H) = −1.2*U*_eq_ (–CH_2_) and *U*_iso_ (H) = −1.2U_eq_ (–CH). Hydrogen atoms on benzene rings were placed in geometric positions via AFIX 43 restriction with the isotropic thermal parameter *U*_iso_ (H) = −1.2U_eq_ (–CH) and refined in a riding model. Hydrogen atoms on the hydroxyl groups of phenolic moieties were found via difference Fourier map inspection and refined with the distance restraint (DFIX O—H 0.84 Å) and with the thermal parameters *U*_iso_ (H) = −1.2*U*_eq_ (–OH). Hydrogen atoms on H_2_O molecules were located via AFIX 7 and refined in a riding model with the isotropic thermal parameters *U*_iso_ (H) = −1.5*U*_eq_ (–OH). Hydrogen atoms on the hydroxyl groups of EtOH and MeOH molecules were located via AFIX 147 and refined in a riding model with the isotropic thermal parameters *U*_iso_ (H) = −1.2*U*_eq_ (–OH). H6 in HESNAH and HESKHE·2H_2_O were refined without any restraint or constraint. A distance restraint DFIX is applied on H1WB and H5, and H1WA and H9A in HESKHE·2H_2_O.

Chiral carbon atoms and neighbouring carbon atoms of HES moieties were modelled for disorder in HESNA·H_2_O without any restraints and constraints applied to C9; in HESNAH·2EtOH with RIGU restraint applied to C8 and C9; in HESK·3H_2_O with distance (SADI) and ADP (ISOR and DELU) restraints applied to C8 and C9; in HESKHE·*x*EtOH with ADP restraints (SIMU, DELU and ISOR) applied to C9, C24 and C25, and with the distance restraint SADI applied on related covalent bonds. One phenolic ring (C26–C31) was modelled for disorder in HESKHE·*x*MeOH. Due to the disordered solvent molecules, HESKHE·*x*MeOH and HESKHE·*x*EtOH were treated by SQUEEZE in *PLATON* (Spek, 2020 ▸), leaving 10.4 and 7.9% void volumes, respectively.

Crystal data have been deposited with the Cambridge Crystallographic Data Centre (CCDC codes 2208281–2208287). Selected crystallographic data and refinement parameters for the crystal structures are given in Table 1 ▸.

### 2.8. Cambridge Structural Database analysis

A Cambridge Structural Database (CSD) survey using *ConQuest* was conducted to search for the distribution of Na/K—O bond lengths with the following constraints: *R*_1_ ≤ 0.05; only organics with 3D coordinates determined from SCXRD; without errors and disorder; Na^+^ or K^+^ cations coordinated to oxygen atoms only; excluding F, Cl, Br, I, S, As, N, P, C, Si and H atoms; coordination number unspecified; bond type between sodium or potassium and oxygen atoms set to ‘any’.

### 2.9. Accelerated stability test

Two scalable ICCs of HES (HESNAH and HESKHE·*x*EtOH) were subjected to an accelerated stability test in a humidity chamber at 40°C and 75% relative humidity (RH) (Huynh-Ba, 2008 ▸). Samples were removed after 14 days and PXRD and TGA data were then collected.

### 2.10. Powder dissolution tests

The dissolution experiments for pure HES and HESNAH were performed in 500 ml of pH 6.8 phosphate buffer solution (PBS) at 37°C under non-sink conditions. A mass of 60 mg equivalent of HES was sieved to 80–106 µm using a standard-mesh sieve and added to 500 ml PBS solution with stirring at 100 rpm. 1 ml aliquots were taken at 2, 4, 6, 8, 10, 15, 30, 40, 60, 90, 120, 150, 180, 210, 240, 270 and 300 min. Each aliquot was filtered through a 0.45 µm Corning syringe filter. 500 µl of filtered aliquot and 500 µl MeOH were added to a vial and injected into the high-performance liquid chromatography (HPLC) instrument. The remaining undissolved solid was analysed by PXRD and TGA. All dissolution experiments were carried out in triplicate.

### 2.11. High-performance liquid chromatography

The dissolution aliquots were analysed using a Shimadzu (LC-20A) HPLC instrument with the Gemini C18 (250 × 4.6 × 5 µm) column. The wavelength was set to 235 nm, the injection volume was set to 5 µl with a flow rate of 1 ml min^−1^ and the oven was set at 40°C. The mobile phase started with a composition of 25%:75% of 0.1% orthophospho­ric acid in aceto­nitrile and 0.1% orthophospho­ric acid in H_2_O with a gradient increase to 100% of 0.1% orthophospho­ric acid in aceto­nitrile from 0 to 13 min, and was then held at 100% for 2 min and afterwards reduced back to 25%:75% over a 2 min gradient. This composition was held constant for a further 3 min.

## 3. Results and discussion

### 3.1. Solid form screening of HES

Fig. 1 ▸ illustrates the crystalline forms of HES reported here. With respect to reactions of HES with sodium methoxide, one salt (HESNA·H_2_O) and two ICCs (HESNAH and HESNAH·2EtOH) were isolated as single crystals suitable for characterization by SCXRD. Attempts were made to scale up all three solid forms. HESNA·H_2_O was isolated from MeOH/*n*-hexane liquid diffusion while attempting to synthesize ICCs. Subsequent attempts to repeat the experiment or to prepare the bulk powder were unsuccessful, affording HESNAH according to PXRD and SCXRD characterization. The same issue was also encountered for scale-up of HESNAH·2EtOH. HESNAH was the only form that could be readily scaled up. This was achieved via slurrying using H_2_O, MeOH or EtOH. In addition, no phase transformation was observed when HESNAH was exposed to accelerated stability testing conditions (75% RH, 40°C) for 14 days as advised by PXRD and TGA (Fig. S1 of the supporting information). Vt-PXRD suggested HESNAH retained its structure up to 280°C (Fig. S2). Therefore, only HESNAH was deemed suitable for dissolution studies.

With respect to reactions of HES with potassium hydroxide, one salt (HESK·3H_2_O) and three ICCs (HESKHE·2H_2_O, HESKHE·*x*MeOH and HESKHE·*x*EtOH) were isolated as single crystals suitable for characterization by SCXRD. One ICC (HESKHE) was obtained as a microcrystalline powder by dehydration of HESKHE·2H_2_O at 160°C (dehydration evidenced by TGA and DSC data; Fig. S3). PXRD patterns [Fig. S4(*a*)] of HESKHE and HESKHE·2H_2_O are different. HESKHE was observed to transform back to HESKHE·2H_2_O after exposure to humidity for one day as supported by TGA [Fig. S4(*b*)], which revealed that transformations between HESKHE and HESKHE·2H_2_O are reversible. Attempts were made to scale up the salt and ICCs. Single crystals of HESK·3H_2_O were isolated in H_2_O using a heating–cooling method but this could not be reproduced. For the ICCs, only bulk preparation of HESKHE·*x*EtOH was successful via EtOH slurry. Unfortunately, HESKHE·*x*EtOH exhibited different TGA features after exposure to humidity for 14 days, likely due to water absorption on the sample surface and EtOH removal from the structure (Fig. S1). In addition, multiple phase changes were observed in vt-PXRD (Fig. S2) after heating to 160°C, which could be attributed to desolvation and/or dissociation of HESKHE·*x*EtOH. Therefore, HESKHE·*x*EtOH was deemed to be unsuitable for dissolution studies.

### 3.2. Crystal structure description

**Figure 2:**
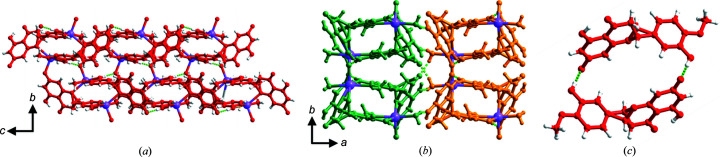
Crystal structure of HESNA·H_2_O. (*a*) and (*b*) Bilayered 2D coordination networks propagate along the crystallographic *bc* plane and are cross-linked by hydrogen bonds between H_2_O molecules and HES^−^ anions along the *a* axis. (*c*) Cyclic dimers of HES^−^ form through pairs of PhOH⋯PhO^−^ hydrogen bonds (motif I) which exist throughout the 2D coordination networks. HES^−^ anions are shown in red.

HESNA·H_2_O (C_16_H_13_O_6_^−^·Na^+^·H_2_O) is a hydrated salt that crystallizes in the space group *P*2_1_/*c* with one Na^+^ cation, one HES^−^ anion and one H_2_O molecule in the asymmetric unit. Each Na^+^ cation is five coordinated to —OH, —OCH_3_, —O— and PhO^−^ groups from three HES^−^ anions and by one H_2_O molecule which acts as terminal aqua ligand (for Na—O bond lengths see Table S1 of the supporting information). Na^+^ cations cross-link HES^−^ anions into a 2D coordination network along the *bc* crystallographic plane [Fig. 2 ▸(*a*)], which comprises side-by-side enclosed ‘squares’ when viewed down the *c* axis, as illustrated in Fig. 2 ▸(*b*), revealing a bilayer 2D coordination network. Along the *a* axis, adjacent 2D polymeric sheets are stacked and interact via hydrogen bonds between H_2_O molecules and HES^−^ anions [Fig. 2 ▸(*b*)]. Hydrogen bonds between HES^−^ anions sustain the 2D coordination networks. HES^−^ anions were found to organize in cyclic dimers through pairs of charge-assisted PhOH⋯PhO^−^ hydrogen bonds (O2⋯O6^−^: 2.526 (2) Å) [Fig. 2 ▸(*c*)], termed ‘motif I’ herein. Further discussion about the interactions between HES^−^ anions is presented below.

**Figure 3:**
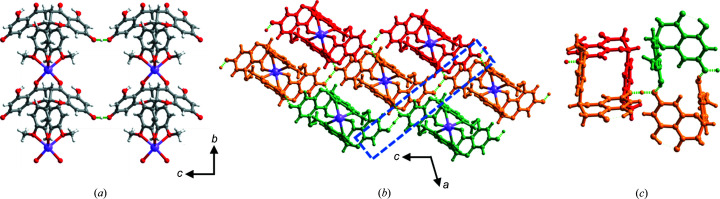
Crystal structure of HESNAH. (*a*) Coordination polymer chains propagate along the *b* axis, giving rise to a 2D network through [PhO⋯H⋯PhO]^−^ hydrogen bonds along the crystallographic *bc* plane. (*b*) Packing of 2D hydrogen-bonding networks in a way that (*c*) HES moieties self-assemble into cyclic dimers through pairs of PhOH⋯PhO^−^ hydrogen bonds (motif I).

HESNAH (C_16_H_14_O_6_·C_16_H_13_O_6_^−^·Na^+^) is an anhydrous ICC that crystallizes in the space group *P*2/*n*. The asymmetric unit contains half the chemical formula (*Z*′ = 0.5) since Na^+^ cations and the protons between HES moieties are located on a crystallographic twofold axis. A symmetric or close-to-symmetric charge-assisted [PhO⋯H⋯PhO]^−^ hydrogen bond is formed with a short O6⋯O6′ distance of 2.4618 (18) Å, the proton was located via difference Fourier map inspection (Kreevoy *et al.*, 1998 ▸). Each Na^+^ cation is six-coordinate through two —OH, two —OCH_3_ and two —C=O groups from four HES moieties (for Na—O bond lengths see Table S1), forming coordination polymer chains that propagate along the *b* axis and stack along the *a* and *c* axes [Figs. 3 ▸(*a*) and 3(*b*)]. The resulting 3D network is sustained by hydrogen bonds. Like HESNA·H_2_O, motif I cyclic dimers form through pairs of hydrogen bonds between phenolate groups (partly deprotonated in HESNAH) and phenolic groups of HES moieties (O2⋯O6 2.636 (2) Å). A chain of cyclic dimers connected by [PhO⋯H⋯PhO]^−^ hydrogen bonds [Fig. 3 ▸(*c*)] is thereby generated. Fig. 3 ▸(*c*) reveals that HES moieties in HESNAH are folded into a ‘V’ shape with a dihedral angle between the benzopyrone rings and phenolic rings of 89.89°. A conformational comparison of HES is addressed below.

**Figure 4:**
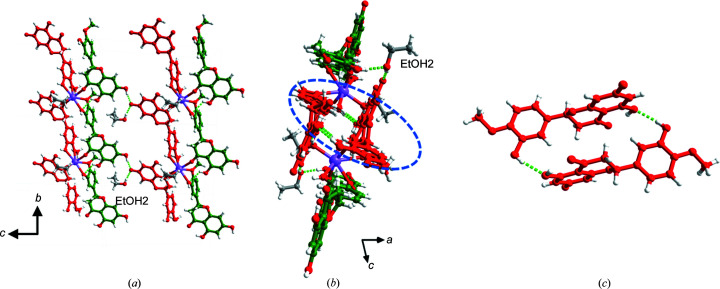
Crystal structure of HESNAH·2EtOH. (*a*) Coordination polymer chains propagate along the *b* axis with HES and HES^−^ arranged on each side and linked to neighbouring chains along the *c* axis via PhOH⋯PhO^−^ hydrogen bonds. (*b*) Adjacent chains along the *a* axis are connected by EtOH molecules and by (*c*) cyclic dimers of HES^−^ anions through pairs of PhOH⋯PhOH hydrogen bonds (motif II). HES molecules and HES^−^ anions are show in green and red, respectively.

HESNAH·2EtOH (C_16_H_14_O_6_·C_16_H_13_O_6_^−^·Na^+^·2EtOH) crystallizes in the space group *P* 1, with HES^−^ anions, Na^+^ cations and HES molecules in a 1:1:1 ratio and two EtOH solvate molecules. Na^+^ cations are seven-coordinate through bonding to HES and HES^−^ via two —OH, two —OCH_3_, two —C=O groups and an EtOH molecule (EtOH1) which acts as a terminal ligand (for Na—O bond lengths see Table S1), resulting in infinite polymer chains propagating along the *b* axis [Fig. 4 ▸(*a*)]. HES molecules and HES^−^ anions arrange on each side of the polymer chains. PhOH⋯PhOH hydrogen bonds (O5⋯O2: 2.945 (3) Å) between HES molecules are present in the polymer chains. Along the *c* axis, adjacent chains are related to each other by PhOH⋯PhO^−^ hydrogen bonds (O6⋯O7^−^: 2.566 (3) Å) between HES molecules and HES^−^ anions [Fig. 4 ▸(*a*)]. Along the *a* axis, free EtOH molecules (EtOH2) lie between adjacent chains through EtOH⋯PhO^−^ and EtOH⋯PhOH hydrogen bonds [Fig. 4 ▸(*b*)]. Cyclic dimers formed through pairs of PhOH⋯PhOH hydrogen bonds (O11⋯O8: 2.853 (3) Å) between HES^−^ anions (termed ‘motif II’ herein) from adjacent chains [Fig. 4 ▸(*b*) and 4(*c*)] were observed. Further discussion concerning the interactions between HES^−^ anions is presented below.

**Figure 5:**
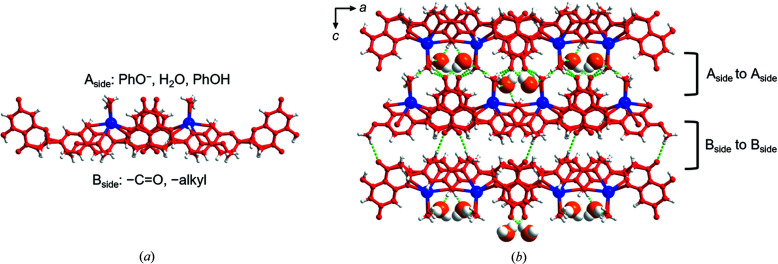
Crystal structure of HESK·3H_2_O. (*a*) 2D coordination network with different functional groups located on two sides, *i.e.* the A_side_ and B_side_. (*b*) Packing of the coordination polymer layers alternating in an ‘A_side_-to-A_side_’ and ‘B_side_-to-B_side_’ fashion; non-coordinated H_2_O molecules (space-filling mode, oxygen atoms are orange) lie between layers in the ‘A_side_-to-A_side_’ region. HES^−^ anions are shown in red.

**Figure 6:**
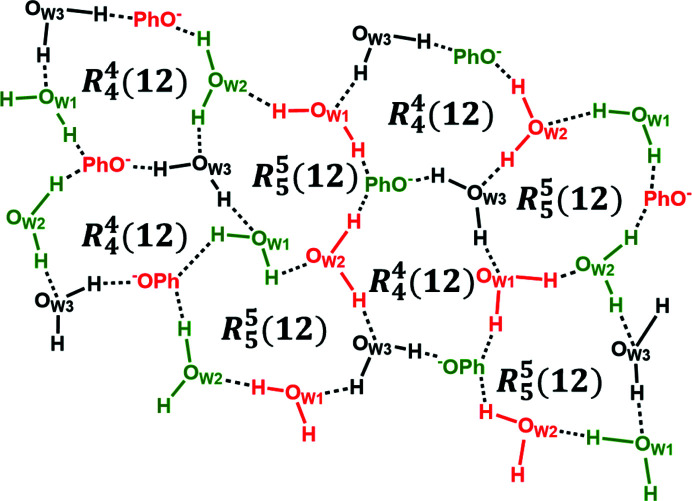
A portion of the hydrogen-bonding network comprised of 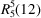 and 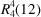 hydrogen-bonded motifs in HESK·3H_2_O. Red and green indicate different layers; non-coordinated H_2_O molecules are shown in black.

HESK·3H_2_O (C_16_H_13_O_6_^−^·K^+^·3H_2_O) is a salt hydrate that crystallizes in the space group *Pbca* with one HES^−^ anion, one K^+^ cation and three H_2_O molecules in the asymmetric unit. Each K^+^ cation is coordinated to six neighbouring oxygen atoms from three HES^−^ anions, including —OH and —OCH_3_ groups on the phenolic rings of one HES^−^, the —OH group on the benzopyrone ring of the second HES^−^, —O— moieties of the third HES^−^ and two H_2_O molecules [H_2_O(1), H_2_O(2)] (for K—O bond lengths see Table S1). K^+^ cations cross-link HES^−^ anions into a 2D coordination network along the *ac* crystallographic plane. When viewed down the *b* axis, all phenolate and phenolic groups of HES^−^ and coordinated H_2_O molecules are positioned on the same side, the A_side_, whereas the B_side_ is occupied by alkyl and —C=O groups, indicating that the A_side_ is rich in hydrogen-bond acceptors and donors whereas the B_side_ is deficient [Fig. 5 ▸(*a*)]. Adjacent coordinated polymer layers stack alternately in an ‘A_side_-to-A_side_’ and ‘B_side_-to-B_side_’ fashion along the *c* axis [Fig. 5 ▸(*b*)]. It is not surprising that only weak hydrogen bonds (*e.g.* C1—H⋯O4) were observed between ‘B_side_-to-B_side_’ layers given the deficiency of hydrogen-bonding points of the B_side_. On the contrary, the ‘A_side_-to-A_side_’ stacking and the presence of free H_2_O molecules between layers facilitate a complex hydrogen-bonding network comprised of two kinds of charge-assisted hydrogen-bonded ring motifs, 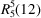 and 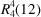 (Fig. 6 ▸). PhOH⋯PhO^−^ hydrogen bonds were not observed in HESK·3H_2_O. Rather, phenolate groups interact with three H_2_O molecules simultaneously.

HESKHE·2H_2_O (C_16_H_14_O_6_·C_16_H_13_O_6_^−^·K^+^·2H_2_O) is a hydrated ICC that crystallizes in the space group *P*2/*n* with half of the formula unit (*Z*′ = 0.5) in the asymmetric unit. Like HESNAH, as a consequence of K^+^ and the proton sitting on a twofold axis, symmetric or close-to-symmetric charge-assisted [PhO⋯H⋯PhO]^−^ hydrogen bonds [O6⋯O6′: 2.451 (2) Å] were observed between HES moieties. Eight coordination is observed around the K^+^ cation, involving oxygen atoms of four HES moieties and two H_2_O molecules which act as terminal aqua ligands (for K—O bond lengths see Table S1). The HES moieties fold perpendicularly between the phenolic ring and benzopyrone ring with a dihedral angle of 86.58°, which is slightly smaller than the 89.89° value found in HESNAH. Therefore, similar coordination polymer chains to those observed in HESNAH propagate along the *b* axis and give rise to a 3D network through hydrogen bonds [Figs. S5(*a*) and S5(*b*)]. The crystal packing is sustained via [PhO⋯H⋯PhO]^−^ and PhOH⋯PhO^−^ hydrogen bonds [O2⋯O6: 2.660 (3) Å] that form cyclic dimers between HES moieties like in HESNAH, and hydrogen bonds between H_2_O molecules and phenolic groups of HES moieties that form 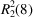 hydrogen-bonded motifs [Figs. S5(*c*) and S5(*d*)]. The similar packing patterns in HESKHE·2H_2_O and HESNAH mean that they can be classified as isostructural (Kálmán *et al.*, 1993 ▸; Wood *et al.*, 2012 ▸) despite the incorporation of H_2_O molecules in HESKHE·2H_2_O and cation substitution. The presence of H_2_O molecules contributes to the larger unit-cell volume [1487.38 (5) Å^3^ HESKHE·2H_2_O; 1373.79 (15) Å^3^ HESNAH]. Despite structural similarities, HESKHE·2H_2_O and HESNAH have distinct PXRD patterns (Fig. S6).

**Figure 7:**
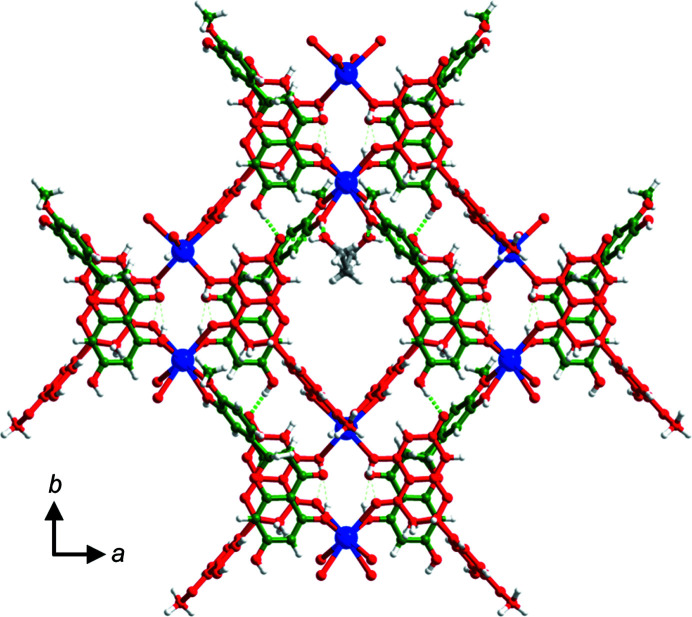
‘Double-wall’ square grid filled with EtOH molecules in the crystal structure of HESKHE·*x*EtOH. HES molecules and HES^−^ anions are shown in green and red, respectively.

HESKHE·*x*EtOH (C_16_H_14_O_6_·C_16_H_13_O_6_^−^·K^+^·*x*EtOH) crystallizes in the space group *C*2/*c*. The asymmetric unit contains two half K^+^ cations, one HES molecule, one HES anion and a non-stoichiometric quantity of EtOH molecules. One K^+^ cation (K1^+^) is coordinated to four —OH and two —OCH_3_ groups of four HES^−^ anions. The second K^+^ cation (K2^+^) is coordinated to four neutral and two anionic HES moieties via four —OH, two —OCH_3_ and two —C=O groups (for K—O bond lengths see Table S1). Their coordination numbers are 6 and 8, respectively. The resulting structure is a ‘double-wall’ square grid propagating in the *ab* crystallographic plane. The square grid is sustained by PhOH⋯PhO^−^ [O6⋯O7^−^: 2.482 (4) Å] and PhOH⋯PhOH hydrogen bonds [O6⋯O11: 2.947 (7) Å] and its cavities are filled with EtOH molecules (Fig. 7 ▸). EtOH molecules interact with HES^−^ through EtOH⋯PhO^−^ hydrogen bonds and with HES though EtOH⋯PhOH hydrogen bonds.

HESKHE·*x*MeOH (C_16_H_14_O_6_·C_16_H_13_O_6_^−^·K^+^·*x*MeOH) and HESKHE·*x*EtOH are isostructural with the same unit-cell parameters and space group except for the replacement of EtOH with MeOH (Fig. S7). The two crystals have PXRD patterns that are substantially the same (Fig. S6).

### 3.3. CSD survey for distribution of Na/K—O bond lengths

**Figure 8:**
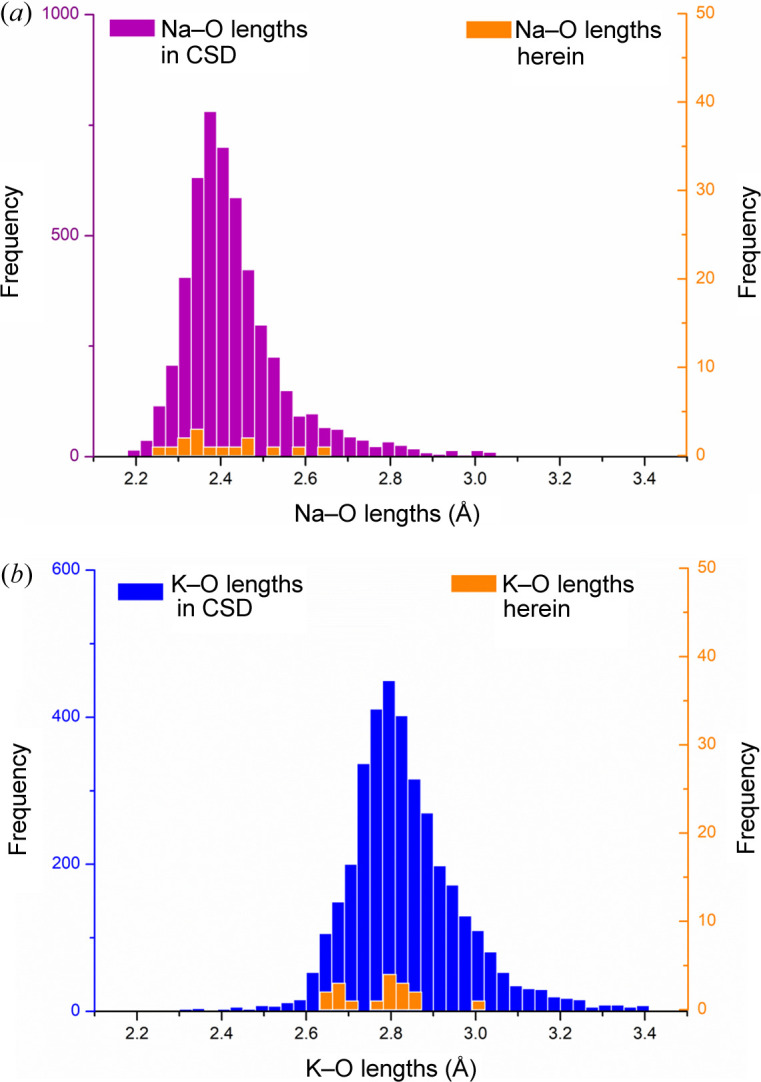
Histograms of (*a*) Na—O and (*b*) K—O bond length distributions. Purple and blue bars represent the bond distances retrieved from the CSD. Orange bars represent the bond distances observed in structures reported here.

A CSD survey resulted in a sample size of 5145 Na—O bond lengths in 666 coordination structures containing sodium. The distribution of Na—O bond distances is presented in Fig. 8 ▸(*a*). Most bond lengths fall in the range 2.1–3.1 Å with a mean value of 2.431 ± 0.127 Å. The CSD survey of K—O bonds resulted in 3692 bond lengths in 462 structures of potassium complexes. The K—O bond length distribution is shown in Fig. 8 ▸(*b*). The majority of K—O bonds exhibit lengths from 2.4 to 3.4 Å, averaging at 2.835 ± 0.137 Å. Na—O and K—O bond lengths extracted from the structures of HES reported here were in the ranges 2.2610 (16) to 2.6371 (15) Å and 2.6450 (19) to 2.8714 (24) Å, respectively (Table S1 and orange bars in Fig. 8 ▸). The bond lengths observed in the structures reported here are therefore in good accordance with the literature values, with Na—O bonds consistently shorter than K—O bonds.

### 3.4. Hydrogen-bonding analysis

In the crystal structures reported here, there are two hydrogen-bond acceptors, *i.e.* C=O and PhOH, that compete with PhO^−^ to form a supramolecular synthon with the hydrogen-bond donor PhOH. According to our CSD analysis, the PhOH⋯PhO^−^ supramolecular synthon occurs in 58.8% of crystal structures that contain PhOH, C=O and PhO^−^ in the absence of COOH or COO^−^ moieties, whereas PhOH⋯PhOH and PhOH⋯O=C synthons occur in 11.8 and 23.5% of related structures, respectively (CSD survey parameters are presented in Table S2). These results indicate that PhOH⋯PhO^−^ supramolecular synthons are generally preferred versus the relevant competing supramolecular synthons. The CSD data are consistent with the results obtained. We note that the structure of HES inherently favours this situation as six-membered-ring intramolecular hydrogen bonds persist between –OH and C=O moieties on the benzopyrone rings of HES, meaning that C=O is less likely to form intermolecular hydrogen bonds with additional PhOH moieties from neighbouring molecules. PhOH⋯PhOH synthons were observed in only three structures: HESNAH·2EtOH, HESKHE·*x*EtOH, HESKHE·*x*MeOH. In contrast, six of the seven crystal structures (except for HESK·3H_2_O) are sustained by PhOH⋯PhO^−^ synthons which exhibit shorter distances [2.451 (2) Å to 2.660 (3) Å] than PhOH⋯PhOH [2.853 (3) Å to 2.947 (7) Å] hydrogen bonds. Table 2 ▸ details the geometric parameters of PhOH⋯PhO^−^ and PhOH⋯PhOH hydrogen bonds observed in the structures reported. PhOH⋯PhO^−^ hydrogen bonds are expected to be stronger as they are charge-assisted as explained through energy decomposition analysis in our previous work (Jin, Sanii *et al.*, 2022 ▸). It has been suggested that symmetric [PhO⋯H⋯PhO]^−^ hydrogen bonds can be classified as quasi-covalent bonds in nature due to incomplete proton transfer (Steiner, 2002 ▸). The [PhO⋯H⋯PhO]^−^ hydrogen bonds observed in HESNAH [2.4618 (18) Å] and HESKHE·2H_2_O [2.451 (2) Å], reported here, HESTEA-γ [2.4256 (19) Å] reported by us recently (Jin, Haskins *et al.*, 2022 ▸), and bis­(4-Nitro­phenoxide) dihydrate [2.434 (6) Å] reported by Kreevoy *et al.* (1998 ▸), are shorter than other PhOH⋯PhO^−^ hydrogen bonds [2.479 (3) Å to 2.660 (3) Å] observed in this work.

HES features multiple phenolic moieties that can participate in hydrogen bonding. Twelve HES entries in the CSD contain HES molecules that form infinite chains via supramolecular homosynthons (PhOH⋯PhOH, five structures) or other supramolecular heterosynthons (PhOH⋯O=C, four structures). The refcodes are summarized in Table S3. No cyclic dimers were found between HES molecules. When HES is deprotonated, the results reported here and in our previous study on polymorphic ICCs of HES with its tetra­ethyl­ammonium salt (Jin, Haskins *et al.*, 2022 ▸) reveal that HES^−^ anions may form infinite chains with (in HESTEA-β) or without (in HESTEA-α, HESTEA-γ) HES molecules. They may also self-assemble into cyclic dimers which were found in four out of the seven structures reported in this work. Motif I cyclic dimers formed by pairs of PhOH⋯PhO^−^ hydrogen bonds exist in HESNA·H_2_O, HESNAH and HESKHE·2H_2_O. Motif II cyclic dimers generated by pairs of PhOH⋯PhOH hydrogen bonds between HES^−^ anions are present in HESNAH·2EtOH. No dimers comprising HES and HES^−^ were observed. It is therefore possible that HES^−^ anions and HES molecules are prone to form infinite chains, while HES^−^ anions can self-assemble into cyclic dimers.

### 3.5. HES conformation analysis

**Figure 9:**
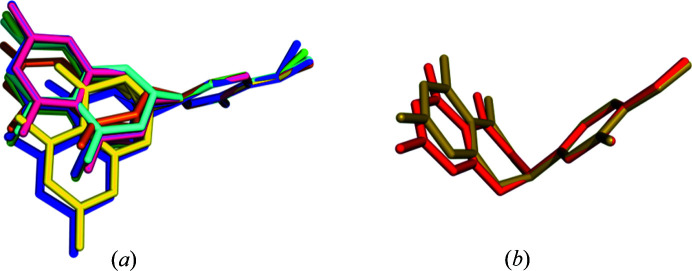
Overlay of (*a*) unfolded conformations of HES moieties found in HESNA·H_2_O (cyan), HESNAH·2EtOH (green for HES^−^, blue for HES), HESK·3H_2_O (orange), HESKHE·*x*MeOH (magenta for HES), HESKHE·*x*EtOH (purple for HES^−^, yellow for HES); and (*b*) folded conformations found in HESNAH (red) and HESKHE·2H_2_O (olive). HES^−^ in HESKHE·*x*MeOH is not included due to phenolic ring disorder. Hydrogen atoms have been omitted for clarity.

HES can exhibit flexibility around its chiral carbon atom, which is evident when the conformations of HES moieties extracted from the structures of HES reported here are overlaid by aligning the phenolic rings (Fig. 9 ▸). Since the torsional angles cannot be determined due to the disorder of the chiral carbon atoms, in general, the conformational variability of HES moieties can be readily assessed through the analysis of dihedral angles between the benzopyrone rings (chiral carbons excluded) and phenolic rings. In HESNA·H_2_O, HESNAH·2EtOH, HESK·3H_2_O, HESKHE·*x*MeOH and HESKHE·*x*EtOH, HES moieties were found to exist in various conformations in such a manner that the benzopyrone rings and phenolic rings are unfolded [Fig. 9 ▸(*a*)]. The dihedral angles in these five structures are given in Table S4 and range from 55.83 to 89.13°. Interestingly, in HESNAH and HESKHE·2H_2_O, HES moieties exhibit conformations in which the benzopyrone and phenolic rings are folded with dihedral angles of 89.89 and 86.58°, respectively [Fig. 9 ▸(*b*)]. By inspection of HES conformations in all structures deposited in the CSD, we found that all HES moieties in the CSD are unfolded as shown in Fig. 9 ▸(*a*); the folded conformation has not been previously reported.

### 3.6. Powder dissolution analysis

**Figure 10:**
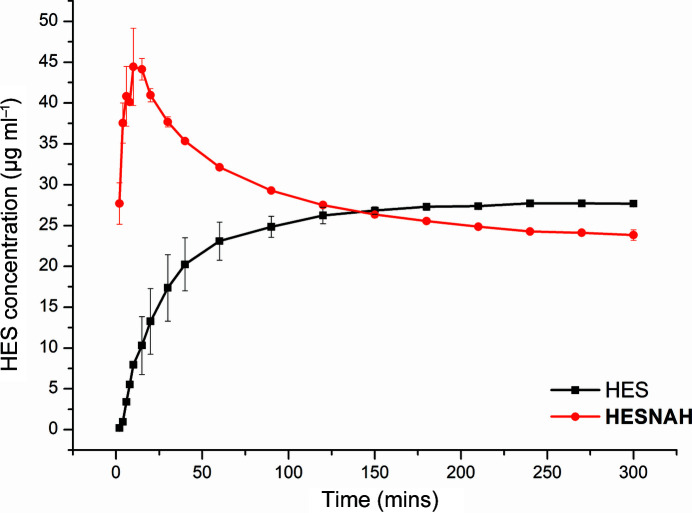
Dissolution profiles of HES and HESNAH in pH 6.8 PBS buffer.

Powder dissolution tests were performed for pure HES and HESNAH. The dissolution profiles are presented in Fig. 10 ▸. Pure HES produced a dissolution profile typical of a poorly soluble molecular compound, demonstrating a slower rate of dissolution before reaching a maximum concentration (*C*_max_) of 27.71 ± 0.30 µg ml^−1^ after 240 min. The residue after dissolution was identified as the anhydrous form of HES (Fig. S8). For HESNAH, an improvement in the HES *C*_max_ was achieved, known as a ‘spring’ effect (Babu & Nangia, 2011 ▸), reaching 44.43 ± 4.74 µg ml^−1^ within 10 min, *i.e.* 5.5 times more than pure HES at 10 min. Subsequently, the concentration dropped to 23.8 ± 0.65 µg ml^−1^, lower than HES bulk solubility, which we attribute to transformation of HESNAH to the hydrated form of HES as indicated by PXRD and TGA (Fig. S8). The fact that HESNAH dissolved at a faster rate compared with pure HES could be beneficial for absorption in the body (Hörter & Dressman, 1997 ▸).

## 4. Conclusions

The present study demonstrates that crystal engineering based on PhOH⋯PhO^−^ supramolecular heterosynthons can be utilized to isolate two new sodium/potassium salts of HES and six ICCs of HES with its conjugate salts. From the structural analysis, we found, as expected, that charge-assisted PhOH⋯PhO^−^ hydrogen bonds are shorter and more linear than the neutral PhOH⋯PhOH hydrogen bonds. Notably, symmetric or close-to-symmetric [PhO⋯H⋯PhO]^−^ hydrogen bonds were observed with short distances. HES moieties were found to exhibit various conformations: unfolded conformations in most structures and folded conformations, we believe for the first time in HES structures, in HESNAH and HESKHE·2H_2_O. Issues relating to scale-up and stability were encountered for both salts and five of the ICCs. HESNAH, however, was found to be scalable and showed a modest improvement in dissolution with respect to pure HES. The improved *in vitro* performance of HESNAH offers the possibility to enhance the pharmacokinetics of HES but requires further study. Overall, the results indicate that, for weakly ionizable compounds such as HES, salt formation may be not an effective route for modulation of physicochemical properties, especially solubility, which is supported by the difficulties we encountered in reproducing the formation of HESNA·H_2_O and HESK·3H_2_O. In addition, the results further support the robustness and reliability of PhOH⋯PhO^−^ supramolecular heterosynthons for crystal engineering of ICCs of phenolic compounds. This is potentially relevant to a broad range of biologically active compounds such as flavonoids and polyphenols.

## Supplementary Material

Crystal structure: contains datablock(s) HESKHE_2H2O, HESKHE_xEtOH, HESKHE_xMeOH, HESK_3H2O, HESNAH, HESNAH_2EtOH, HESNA_H2O. DOI: 10.1107/S205225252300266X/lq5051sup1.cif

Structure factors: contains datablock(s) HESK_3H2O. DOI: 10.1107/S205225252300266X/lq5051HESK_3H2Osup2.hkl

Structure factors: contains datablock(s) HESKHE_2H2O. DOI: 10.1107/S205225252300266X/lq5051HESKHE_2H2Osup3.hkl

Structure factors: contains datablock(s) HESKHE_xEtOH. DOI: 10.1107/S205225252300266X/lq5051HESKHE_xEtOHsup4.hkl

Structure factors: contains datablock(s) HESKHE_xMeOH. DOI: 10.1107/S205225252300266X/lq5051HESKHE_xMeOHsup5.hkl

Structure factors: contains datablock(s) HESNA_H2O. DOI: 10.1107/S205225252300266X/lq5051HESNA_H2Osup6.hkl

Structure factors: contains datablock(s) HESNAH_2EtOH. DOI: 10.1107/S205225252300266X/lq5051HESNAH_2EtOHsup7.hkl

Structure factors: contains datablock(s) HESNAH. DOI: 10.1107/S205225252300266X/lq5051HESNAHsup8.hkl

Supporting figures and tables. DOI: 10.1107/S205225252300266X/lq5051sup9.pdf

CCDC references: 2208281, 2208282, 2208283, 2208284, 2208285, 2208286, 2208287

## Figures and Tables

**Table 1:** Crystallographic data and structure refinement parameters Hydrogen atoms were treated by a mixture of independent and constrained refinement.

	HESNA·H_2_O	HESNAH	HESNAH·2EtOH	HESK·3H_2_O	HESKHE·2H_2_O	HESKHE·*x*MeOH	HESKHE·*x*EtOH
Crystal data							
Chemical formula	C_16_H_13_O_6_Na·H_2_O	C_32_H_27_O_12_Na	C_32_H_27_O_12_Na·2C_2_H_6_O	C_16_H_13_O_6_K·3H_2_O	C_32_H_27_O_12_K·2H_2_O	C_32_H_27_O_12_K·*x*CH_4_O	C_32_H_27_O_12_K·*x*C_2_H_6_O
*M*_r_	342.27	313.26	718.66	394.41	678.67	674.68	688.70
Crystal system	Monoclinic	Monoclinic	Triclinic	Orthorhombic	Monoclinic	Monoclinic	Monoclinic
Space group	*P*2_1_/*c*	*P*2/*n*	*P* 1	*Pbca*	*P*2/*n*	*C*2/*c*	*C*2/*c*
T (K)	150	150	150	113	116	113	113
*a* (Å)	11.0666 (4)	10.7741 (6)	10.8865 (5)	19.9343 (3)	11.2185 (2)	19.6211 (3)	19.4072 (8)
*b* (Å)	13.2714 (4)	9.6357 (6)	10.9884 (6)	7.8657 (2)	10.2204 (2)	20.3628 (3)	20.4384 (8)
*c* (Å)	10.0412 (3)	13.8452 (9)	16.1451 (8)	31.4425 (7)	13.5244 (2)	17.2684 (3)	17.3645 (11)
α (°)	90	90	81.871 (4)	90	90	90	90
β (°)	94.430 (1)	107.103 (2)	74.681 (5)	90	106.427 (1)	104.282 (1)	104.006 (5)
γ (°)	90	90	68.049 (5)	90	90	90	90
*V* (Å^3^)	1470.34 (8)	1373.79 (15)	1725.73 (17)	3446.19 (14)	1487.38 (5)	6686.19 (19)	6682.9 (6)
*Z*, *Z*′	4, 1	2, 0.5	2, 1	8, 1	2, 0.5	8, 1	8, 1
Radiation type	Mo *K*α	Mo *K*α	Mo *K*α	Cu *K*α	Cu *K*α	Cu *K*α	Mo *K*α
μ (mm^−1^)	0.15	0.13	0.12	3.15	2.23	1.96	0.23
Diffractometer	Bruker APEX-II CCD	Bruker APEX-II CCD	Rigaku mm007 Oxford	Bruker APEX-II CCD	Bruker APEX-II CCD	Bruker APEX-II CCD	Rigaku mm007 Oxford
*T*_min_, *T*_max_	0.710, 0.746	0.715, 0.746	0.904, 1.000	0.542, 0.753	0.509, 0.753	0.594, 0.753	0.373, 1.000
Measured [*I* > 2σ(*I*)] reflections	14984	13484	18542	29419	14054	39471	26486
Independent [*I* > 2σ(*I*)] reflections	3368	3173	7061	3040	2610	5880	6841
Observed [*I* > 2σ(*I*)] reflections	2734	2484	5100	2903	2414	5781	5112
*R*_int_	0.024	0.045	0.047	0.071	0.049	0.040	0.062

Refinement							
*R*[*F* ^2^ > 2σ(*F* ^2^)]	0.048	0.043	0.061	0.045	0.054	0.055	0.080
w*R*(*F* ^2^)	0.124	0.117	0.165	0.117	0.161	0.147	0.205
*S*	1.04	1.07	1.05	1.08	1.07	1.13	1.02
Reflections	3368	3173	7061	3040	2610	5880	6841
Parameters	234	212	501	267	222	541	485
Restraints	2	2	11	19	4	405	52

**Table 2:** PhOH⋯PhO^−^ and PhOH⋯PhOH hydrogen-bond parameters observed in the salt and ICC structures of HES reported here

Solid forms	*d* (*D*—H) (Å)	*d* (H⋯*A*) (Å)	*D* (*D*⋯*A*) (Å)	θ (°)
PhOH⋯PhO^−^ hydrogen bonds				
HESNA·H_2_O	0.852 (19)	1.674 (19)	2.526 (2)	180 (3)
HESNAH	0.86 (2)	1.79 (2)	2.636 (2)	165 (2)
	1.2312 (13)	1.2312 (13)	2.4618 (18)	178 (2)
HESNAH·2EtOH	0.87 (2)	1.70 (2)	2.566 (3)	173 (3)
HESKHE·2H_2_O	0.82 (2)	1.84 (2)	2.660 (3)	174 (3)
	1.225 (2)	1.225 (2)	2.451 (2)	180 (5)
HESKHE·*x*MeOH	0.84	1.65	2.479 (3)	169
HESKHE·*x*EtOH	0.84 (4)	1.66 (4)	2.482 (4)	164 (5)

PhOH⋯PhOH hydrogen bonds				
HESNAH·2EtOH	0.85 (2)	2.47 (3)	2.945 (3)	116 (3)
	0.83 (3)	2.04 (3)	2.853 (3)	165 (3)
HESKHE·*x*MeOH	0.85 (6)	2.02 (6)	2.872 (4)	178 (10)
HESKHE·*x*EtOH	0.87 (6)	2.21 (6)	2.947 (7)	143 (4)
