# Supplementary material for: Crystal engineering of ionic cocrystals comprising Na/K salts of hesperetin with hesperetin molecules and solubility modulation
Source: IUCrJ. 2023 Apr 21;10(Pt 3):329–40. doi: 10.1107/S205225252300266X (PMC10161764; doi:10.1107/S205225252300266X)
Supplement: Supplementary file 9 [file m-10-00329-sup9.pdf]

IUCrJ

Volume 10 (2023)

Supporting information for article:

Crystal engineering of ionic cocrystals comprising Na/K salts of hesperetin with hesperetin molecules and solubility modulation

Shasha Jin, Molly M. Haskins, Cheng-Hua Deng, Catiúcia R. M. O. Matos and Michael J. Zaworotko

Table S1 K/Na−O bond lengths observed in the structures of HESNAO and HESKOH.

Solid forms	Coordination number	Coordination Network	Distance Å
**Na−O bond lengths in HESNAO**			
**HESNA·H₂O**	5	2D	2.3320(17)<br>2.3792(15)<br>2.6371(15)<br>2.2610(16)<br>2.3222(16)
**HESNAH**	6	1D	2.3361(15)<br>2.4399(15)<br>2.4039(13)
**HESNAH·2EtOH**	7	1D	2.4669(21)<br>2.4557(19)<br>2.5960(17)<br>2.5267(24)<br>2.2885(22)<br>2.3117(22)<br>2.3395(24)
**K−O bond lengths in HESKOH**			
**HESK·3H₂O**	6	2D	2.6450(19)<br>3.0171(17)<br>2.7970(17)<br>2.7653(16)<br>2.7911(17)<br>2.800(17)
**HESKHE·2H₂O**	8	1D	2.6541(22)<br>2.8166(20)<br>2.8050(19)<br>2.8218(23)
**HESKHE·xEtOH**	6, 8	2D	2.8237(85)<br>2.8647(37)<br>2.7121(36)<br>2.6676(30)<br>2.6712(26)<br>2.6637(34)<br>2.8430(27)
**HESKHE·xMeOH**	6, 8	2D	2.8548(19)<br>2.6593(22)

2.6587(16)
2.6813(22)
2.7434(21)
2.8714(24)

*one K−O bond length in **HESKHE·xMeOH** is not included due to oxygen atom disorder.

Table S2 Frequency of PhOH···PhO¯, PhOH···PhOH, and PhOH···O=C supramolecular synthons appearing in related structures deposited in CSD. (ConQuest version 2020.3.0 with Nov. 2022 update, search parameters: 3D coordinates present; only organics; R factor ≤ 0.05, no disorder and single-crystal structure only).

REFCODEs of structures deposited in CSD that contain PhOH, PhO¯, and O=C moieties with no COOH or COO¯ moieties					
COLHER	DAVLER	DUCWUV	FILGUF	HEGHEH	HIVBET
JICKIS	JIYXEX	LUZRAZ02	NICCEJ	SAFMOF	UNIHUU
UYOYIR	WIHFAW	IWUGEP	XAZHIT	XAZHOZ	

PhOH···PhO¯, PhOH···PhOH, PhOH···O=C, and other supramolecular synthons occur in 58.8%, 11.8%, 23.5%, and 29.4% of structures, respectively.

Table S3 HES entries in the CSD where infinite chains of HES molecules form via either PhOH···PhOH or PhOH···O=C supramolecular synthons.

HES Chains	
**PhOH···PhOH**	**PhOH···O=C**
FOYTOC	IJIWAC
LAVLEC	LAVLAY
RUWHEX	LAVLIG
RUWHIB	YEHROS
CANPOA	

Table S4 Dihedral angles between benzopyrone rings and phenolic rings of HES moieties extracted from structures of HES reported herein.

Solid forms	Dihedral angles/°
**HESNA·H_2O**	75.11
**HESNAH·2EtOH**	86.94, 82.01
**HESK·3H_2O**	55.83
**HESKHE·xEtOH**	81.47, 89.13
**HESKHE·xMeOH**	88.67
**HESNAH**	89.89*
**HESKHE·2H_2O**	86.58*

*folded conformation

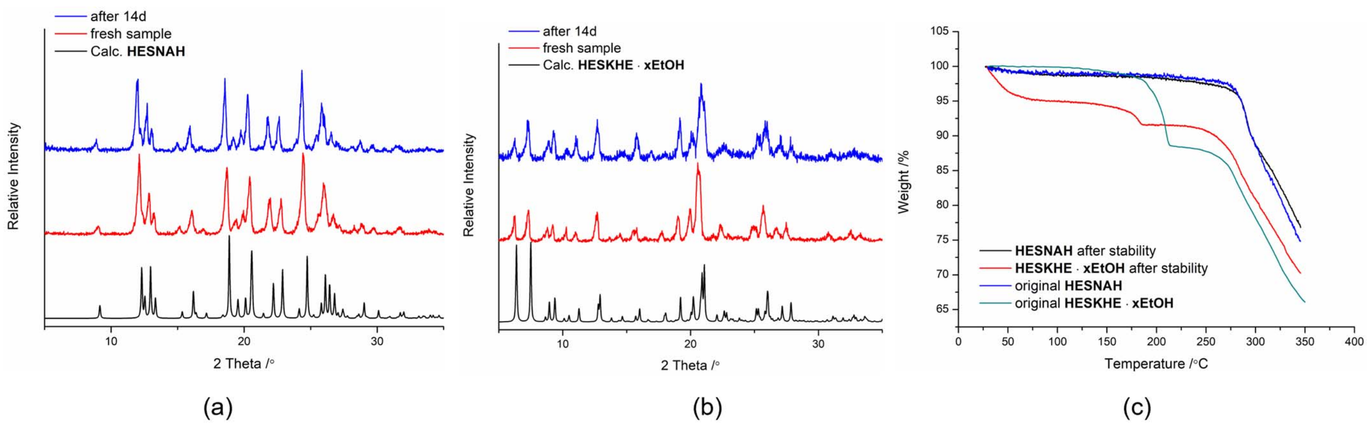

Figure S1 (a) (b) PXRD patterns and (c) TGA plots of **HESNAH** and **HESKHE·xEtOH** after 14 days in accelerated stability test (there is peak shift due to thermal expansion as ICCs were collected at low temperature).

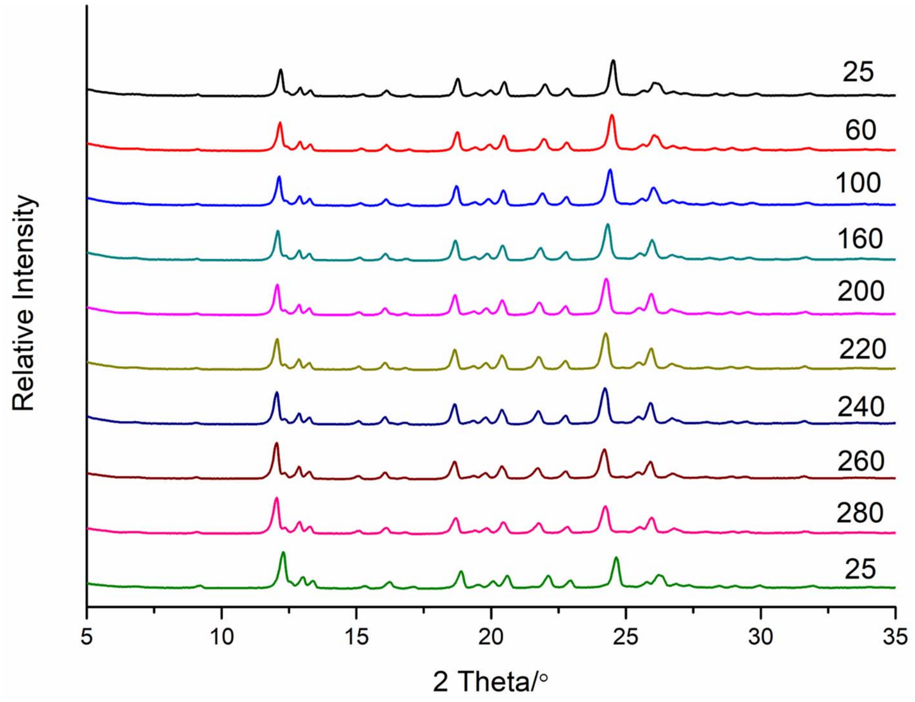

(a)

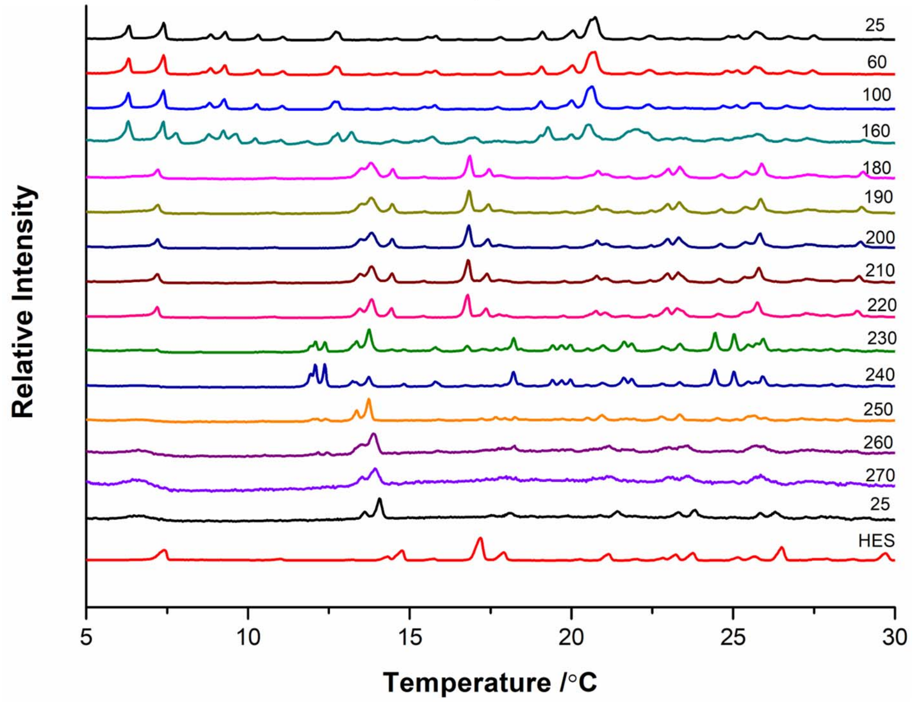

(b)

Figure S2 Variable temperature PXRD of (a) **HESNAH** from 25 °C to 280 °C and back to 25 °C which retained the structure until 280 °C; and (b) **HESKHE·xEtOH** from 25 °C to 270 °C and back to 25 °C. **HESKHE·xEtOH** remined unchanged before desolvation at *ca.* 160 °C confirmed by TGA in Figure S3, and then changed to a phase displaying similar PXRD to HES until 220 °C (likely caused by dissociation to HES which is supported by DSC displaying HES melting peak at similar temperature) followed by another phase change during 230 - 240 °C and then decomposition after 250

°C as black decomposed compound was observed after test (there is peak shift due to thermal expansion as ICC samples were measured at different temperature).

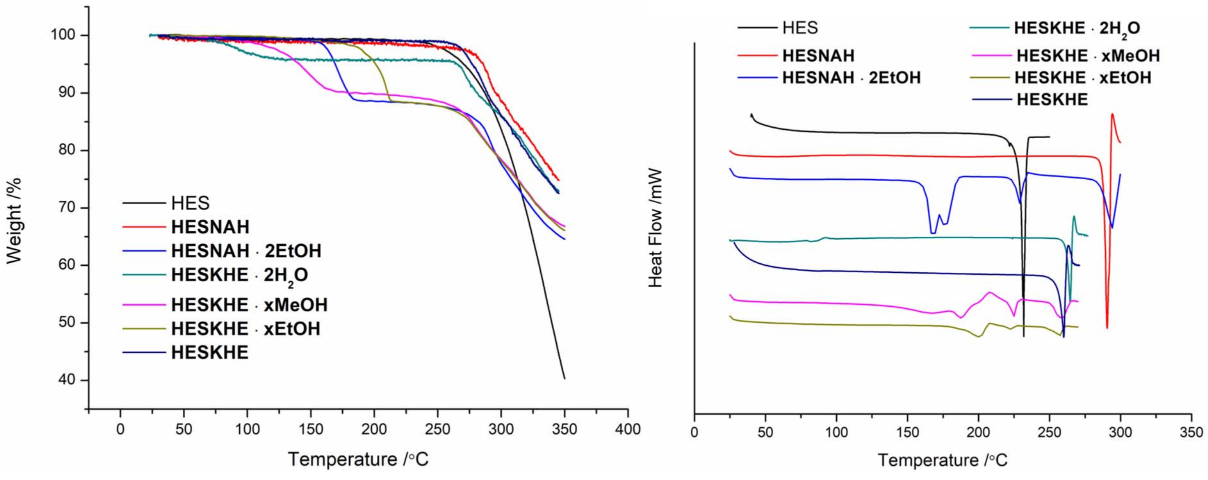

(a) (b)

Figure S3 TGA (a) and DSC (b) plots of HES and ICCs. The weight losses in **HESKHE·xMeOH** and **HESKHE·xEtOH** samples calculated from TGA curves are 9.8% and 11.6%, respectively, which indicates 1.75~2.2 solvent molecules in both structure formulas.

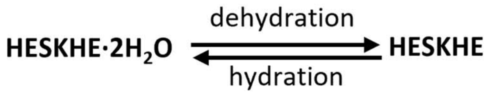

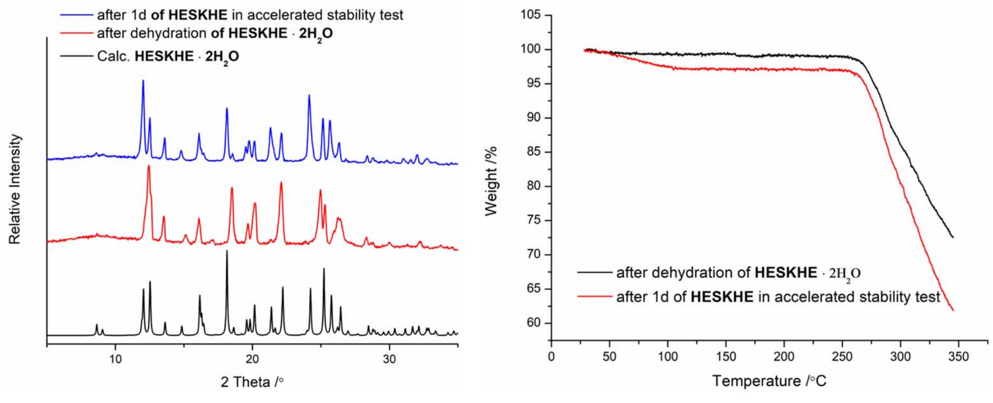

(a) (b)

Figure S4 (a) PXRD patterns and (b) TGA plot of dehydration of **HESKHE·2H₂O** and hydration of **HESKHE** exposed to 75% RH for one day.

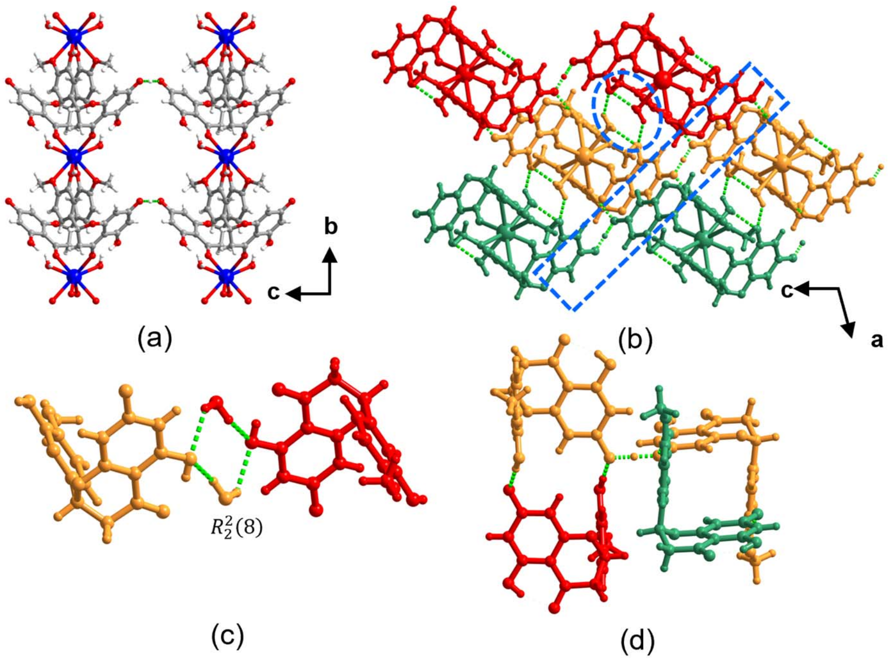

Figure S5 Crystal structure of **HESKHE·2H₂O**. (a) Coordination polymeric chains give rise to 2D network via [PhO···H···PhO⁻] H-bonds. (b) Packing of layers (represented in different colors) causing the formation of (c) $R_2^2(8)$ H-bonded motif between HES moieties and H_2O molecules, and (d) a chain of cyclic dimers of HES moieties (Motif I).

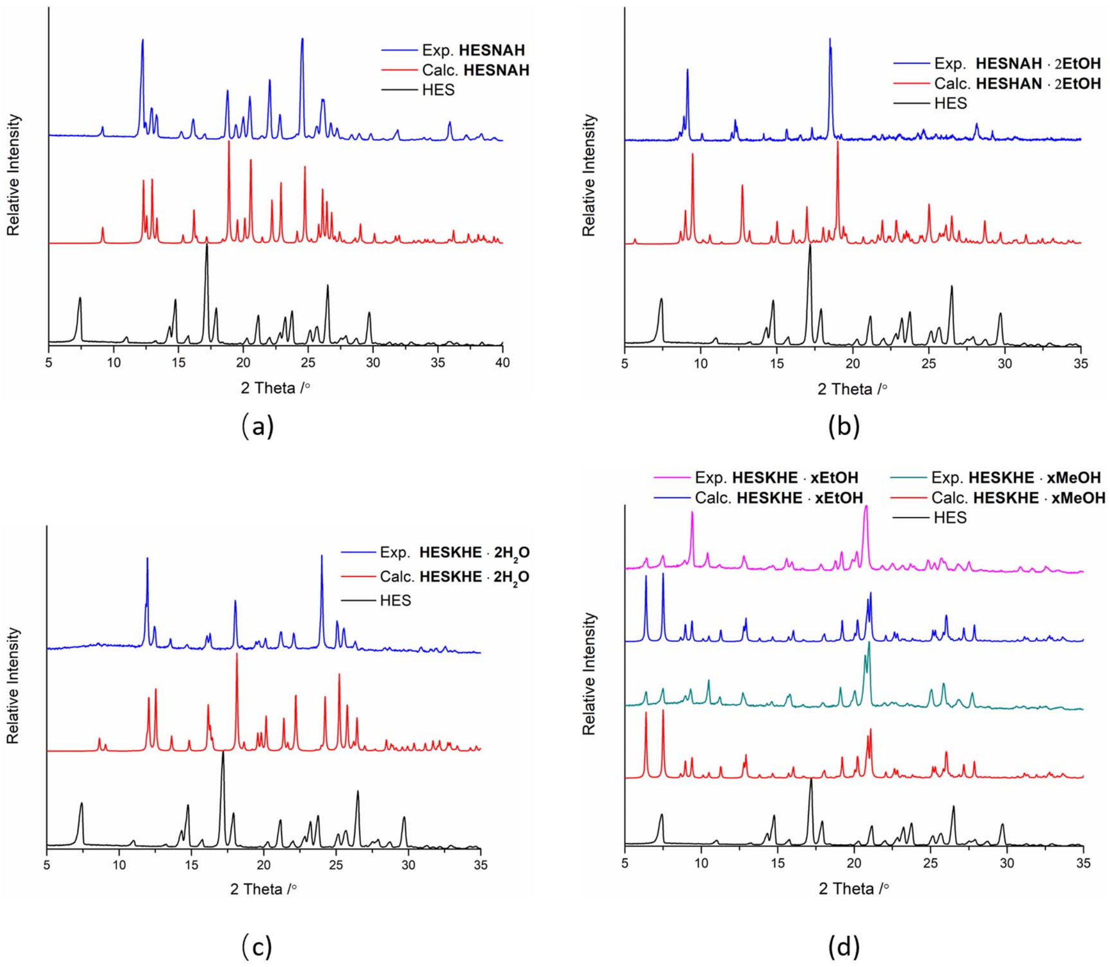

Figure S6 Calculated PXRD generated from single crystal structures of (a) **HESNAH**, (b) **HESNAH·2EtOH**, (c) **HESKHE·2H$_2$O** and (d) **HESKHE·xMeOH** and **HESKHE·xEtOH** compared with experimental PXRD (there is peak shift due to thermal expansion as ICCs were collected at low temperature).

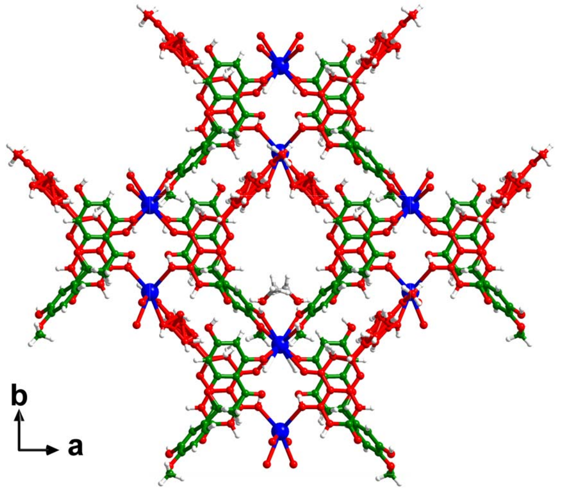

Figure S7 'Double-wall' square grid filled with MeOH molecules in the crystal structure of **HESKHE·xMeOH**. HES molecules and HES⁻ anions are colored green and red, respectively.

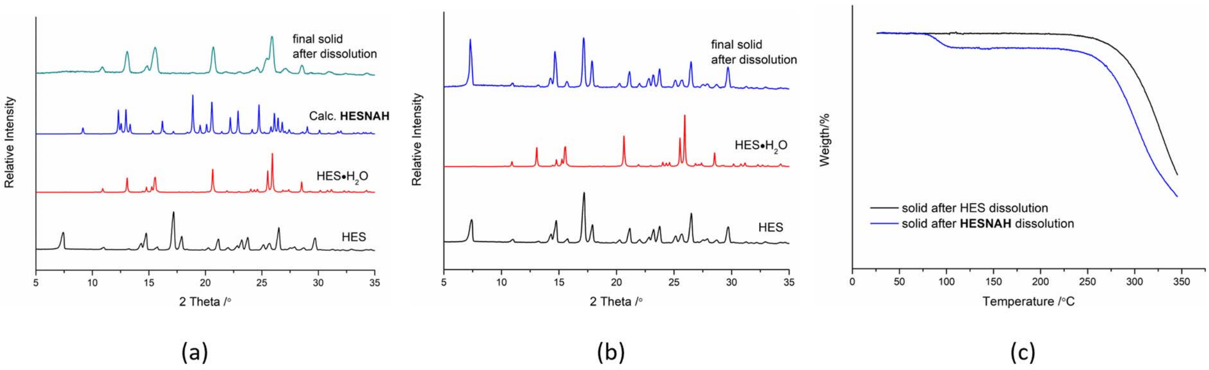

Figure S8 (a) (b) PXRD patterns and (c) TGA plots of final solid forms after dissolution test of **HESNAH** and HES at PBS buffer at pH6.8.
